# Tools/instruments for assessing YouTube videos on surgical procedures for patient/consumer health education: a systematic review

**DOI:** 10.3389/fpubh.2025.1575801

**Published:** 2025-07-10

**Authors:** Manasa Pavuloori, Amy Lin, Misa Mi

**Affiliations:** ^1^Oakland University William Beaumont School of Medicine, Rochester, MI, United States; ^2^Department of Foundational Medical Studies, Oakland University William Beaumont School of Medicine, Rochester, MI, United States

**Keywords:** patient education, surgical procedure, social media, YouTube videos, quality assessment, psychometrics, health education

## Abstract

**Background:**

YouTube is becoming an increasingly popular platform for health education; however, its reliability for surgical patient education remains largely unexplored. Given the global prevalence of preoperative anxiety, it becomes essential to ensure accurate information online.

**Objectives:**

The objective is to assess tools/instruments used to evaluate YouTube videos on surgical procedures created to educate patients or health consumers.

**Methods:**

In June 2023, a comprehensive literature search was conducted on PubMed, PsycINFO, CINAHL, and Scopus. Primary studies with empirical data that evaluate English YouTube videos to educate patients about surgical procedures in all specialties were included. Two reviewers independently completed title/abstract and full text screening, and data extraction in duplicate. The data extracted includes the number of videos evaluated, assessment tools, outcomes of significance, specific objectives, and features examined.

**Results:**

A total of 41 studies were included in the review. The most commonly used evaluation tools were DISCERN (21 studies), the Global Quality Scale (11 studies), and the JAMA benchmark criteria (11 studies). Notably, 23 studies used a unique assessment instrument, and several studies employed more than one tool concurrently. Of the total studies included, 88% of the articles determined that patients were not adequately educated by YouTube videos per the ratings of the assessment tools, and 19 out of 41 articles mentioned that videos from professional sources were most useful.

**Conclusions:**

This systematic review suggests that the educational qualities in YouTube videos are substandard. Patients should be cautious when relying solely on YouTube videos for medical guidance. Surgeons and medical institutions are encouraged to direct patients to high-quality patient education sources and create accessible medical content. As there is variability in the quality assessment tools used for evaluation, a standardized approach to creating and assessing online medical videos would improve patient education.

## Introduction

In 2024, the number of active users on YouTube exceeded 2.56 billion (1). More than 500 h of content are uploaded to YouTube every minute (2). Approximately 25% of adults in the United States stated that they rely on YouTube as a regular source for obtaining news (3).

In recent years, YouTube has emerged as a popular platform for patient and health consumer education. In 2020, 40.8% of U.S. adults used YouTube to watch health–related videos (4). Recent literature published in 2022 has shown that YouTube is not a reliable source for medical and health-related information and there has only been one systematic review investigating the reliability of YouTube as a source of knowledge for surgical patients (5, 6). With the increasing availability of surgical videos on YouTube, it is crucial to assess the tools or instruments used to evaluate the quality and educational value of such content.

Surgical education plays a vital role in empowering patients to make informed decisions about their healthcare and enhance their understanding of complex medical interventions. YouTube offers an easily accessible and visually engaging platform to deliver such educational content. As preoperative anxiety remains a critical issue, occurring in ~48% of surgical patients globally (7), it is vital to ensure that accessible information online on surgical procedures is accurate and regulated to prevent unnecessary confusion.

The purpose of this systematic review is to assess the tools or instruments employed for evaluating YouTube videos focused on surgical procedures with the intent of educating patients or health consumers. The findings of this review will have implications for healthcare providers, educators, and content creators involved in patient education. Understanding the strengths and weaknesses of existing evaluation tools will facilitate the development of standardized guidelines and best practices for assessing the quality and educational impact of YouTube videos on surgical procedures. Ultimately, this systematic review aims to contribute to the improvement of patient education materials available on YouTube, ensuring that patients and health consumers have access to reliable, accurate, and informative content that enhances their surgical knowledge and decision-making abilities.

## Methods

### Literature search

A comprehensive literature search (MM) was conducted using PubMed, PsycINFO, CINAHL and Scopus from each database's inception to June 6, 2023. A combination of index terms and keywords were used to represent key concepts of “patient education,” “YouTube video,” “psychometrics,” “quality assessment,” and “surgical procedure” (See Appendix A for a sample search strategy for PubMed). A hand search of the reference lists of all identified studies were examined for additional studies.

### Eligibility criteria

Articles were selected based on specific inclusion criteria. The review included original, full-text primary studies published in the English language that provided empirical data evaluating English YouTube videos created for patient and health consumer education. Videos encompassed information regarding surgical procedures in all surgical specialties. Reviews, duplicate articles, comments, editorials, letters, and abstracts lacking full content articles were excluded. Studies analyzing videos in other languages, from differing social media sites, and targeting health professional education were also excluded.

### Data selection

All search results were imported into Covidence for screening and data extraction. Covidence is a web-based software for managing and streamlining systematic reviews. Two reviewers (MP and AL) first screened titles and abstracts against the selection criteria, followed by full text screening done in duplicate and independently. Any discrepancies in screening by the two reviewers were discussed and resolved to reach consensus. The third author (MM) assessed any variances and determined their inclusion.

### Data extraction

A standardized data collection form was created on the Covidence platform, and the authors (MP and AL) completed data extraction in duplicate and independently. All discrepancies were discussed and resolved with the third author (MM). The parameters consisted of study aims, surgeries evaluated, type of quality tools used, number of videos analyzed, primary source of videos, types of video characteristics studied, educational quality based on author's judgment, study limitations, future recommendations, and video sources deemed the most useful. The video sources were divided into four categories: commercials, patients, professional entities (e.g., created by physicians, hospitals), educational institutions (association, organization, society, and others). A rating scale was developed to assess the educational quality of the videos: “poor,” “moderate” or “good” based on the articles' direct analyses, and the reasoning was noted.

## Results

**Figure 1:**
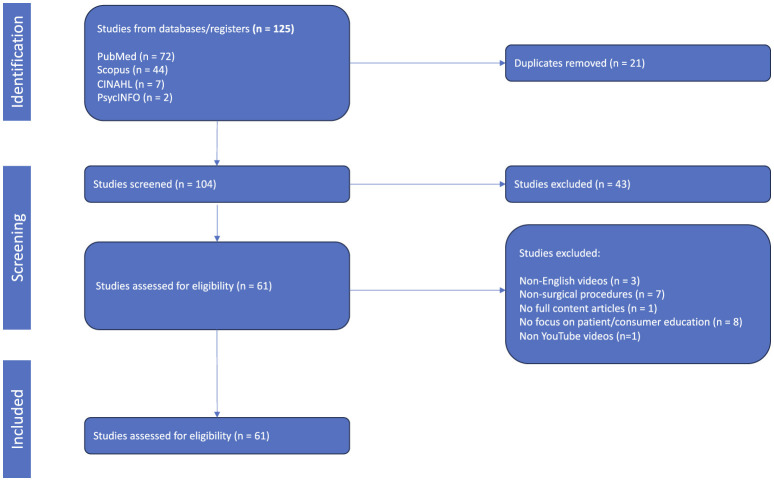
PRISMA flow diagram of article selection process.

Using an initial dataset of 125 studies, articles were excluded based on specific inclusion criteria (Figure 1). Ultimately, 41 studies remained in the review for data extraction and analysis.

### Video information data

**Figure 2:**
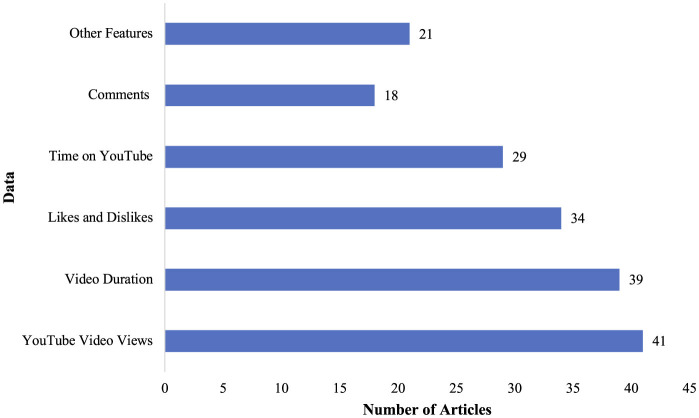
Data assessed by quality assessment tools with “Other Features,” including title of video, universal resource locator, number of channel subscribers, video power index, country of origin, percentage positivity (proportion of likes to total likes plus dislikes), presence of subtitles, viewer interaction index, video title, target audience, presence of animation, video/audio quality, and daily viewing rate.

Using an initial dataset of 125 studies, articles were excluded based on specific inclusion criteria (Figure 1). Ultimately, 41 studies remained in the review for data extraction and analysis. These articles assessed an average of 98.8 videos per study, ranging from 16 to 523 videos evaluated per study. The parameters that quality assessment tools analyzed included YouTube video views, video duration, likes and dislikes, time on YouTube, comments, and other features (Figure 2). Studies were published from 2013 to 2023, with 2021 as the median publication year with a notable increase after 2020. A trend was noted for an increasing amount of research articles evaluating the quality of YouTube videos, reflecting growing reliance on social media and digital platforms for patient education. Videos encompassed information regarding surgical procedures in all surgical specialties, such as general surgery, oral and maxillofacial surgery, cardiac surgery, orthopedic surgery, dental and endodontic surgery, obstetric surgery, gynecology, urology, ophthalmology, neurosurgery, plastic and reconstructive surgery, neonatal surgery, and colorectal surgery.

### Video source characteristics

All of the YouTube videos included professional sources and creators, such as physicians, hospitals, educational institutions, and societies. The majority (68%) featured patients and their testimonials. Commercial content (59%) was present in over half of the videos, while other diverse sources were also frequently utilized (73%). Of the 23 studies reporting sources that provided the most useful data for patient and health consumer education, 20 recognized videos from professional sources, two noted patient sources, and one described commercials as the most helpful source. These findings suggest a strong association between source professionalism and perceived educational value.

### Use of quality assessment tools

The DISCERN reliability instrument was the predominant tool for video evaluation (8–16, 18–29). Other prevalent tools were the Journal of the American Medical Association (JAMA) Benchmark Criteria (8–18), Global Quality Scale (GQS) Criteria (8–10, 14, 17–19, 26, 27, 30, 31), Health on the Net (HON) Code of Conduct (12, 13), Patient Education Materials Assessment Tool (PEMAT) and the Usefulness Scoring System (20, 29). A notable portion of the articles used standardized reliability instruments developed by the authors, from previous studies, physicians and medical organizations (10, 15–17, 27, 30–47). The YouTube Video Assessment Criteria and Ensuring Quality Information for Patients Score were utilized less frequently (Table 1). While standardized tools provided a consistent framework, variation in implementation and scoring limited comparability. Notably, all studies that noted adequate patient education or moderate quality based on article author analysis used multiple assessment tools (e.g., DISCERN + JAMA + GQS).

**Table 1:** Quality assessment tools ranked by frequency of their use across all articles (*n* = 41).

**Type of assessment tool**	**Number of videos**
DISCERN	21
JAMA	11
GQS	11
HON	2
PEMAT	2
Likert 5-point	1
Other	24

### Educational value

Educational quality, as rated by the respective quality assessment instruments, was predominantly low. Based on the articles' analyses of the educational quality of the YouTube videos, 33 studies were rated as poor quality, seven as moderate quality, and one as good quality. Five of the seven studies that determined moderate quality utilized GQS, four used DISCERN, three used JAMA, and four used other types of assessment tools, suggesting some consistency among these tools in identifying informative videos (8–10, 26, 30, 46, 48). The article that reported the videos were of good quality used JAMA, GQS, and its own quality assessment tool as well (17).

Three studies concluded that surgical YouTube videos adequately educated patients, and 36 yielded contrasting data. Many articles (44%) mentioned that YouTube videos were missing vital information regarding surgical procedures, such as treatment alternatives and potential risks. Others (27%) noted that the scores associated with the reliability instruments were low and 20% of the articles saw an increasing prevalence of source bias with the videos. Several studies (15%) detailed that there were notable issues with videography, like difficulties with music, overall quality, and narration. The three other studies noted high scores among the reliability instruments (9), reputable sources creating and distributing content (10), and easily understandable videos with adequate procedure descriptions, which are not systematically captured by existing assessment instruments (8). Interestingly, among the five videos related to ophthalmology, two were reported to have adequately educated patients.

### Common deficiencies in video content

Several recurring deficiencies in YouTube surgical video content were identified across the included studies. Most notably, eighteen studies (44%) reported that videos often omitted critical information such as treatment alternatives, potential risks, or post-operative expectations, limiting their utility for comprehensive patient education. Additionally, eleven studies (27%) documented consistently low scores across validated reliability instruments, including DISCERN and JAMA, reflecting concerns about content accuracy and trustworthiness. Eight studies (20%) highlighted the presence of source bias or overt promotional messaging, which may compromise the objectivity of the information presented. Furthermore, six studies (15%) described production-related limitations, such as poor narration quality, distracting background music, or inadequate video resolution, which could detract from viewer comprehension.

## Discussion

Patient and health consumer education is rapidly evolving, with digital platforms and social media resources becoming increasingly prominent. This shift requires a reevaluation of how surgical patients receive information about their conditions and procedures. Validated evaluation tools are essential for assessing the quality and accuracy of educational videos to identify videos that provide clear information and align with current medical standards.

### Video sources and content

Since all 41 articles evaluated YouTube videos that included professional sources, the content is likely to be credible and created with expertise. However, despite this professional endorsement, the majority of the videos were deemed to provide poor educational quality. The prevalence of patient testimonials and commercial content contributes to further complications as these types of videos often prioritize personal experiences and promotional content over comprehensive educational information.

### Quality assessment tools and quality analysis

To assess video quality, the authors predominantly utilized the DISCERN reliability instrument, along with other established tools such as the JAMA Benchmark Criteria and the GQS Criteria.

The DISCERN tool is a validated questionnaire designed to assess the quality of written consumer health information on treatment choices, consisting of 15 items rated on a 5-point scale and culminating in an overall quality score. Though comprehensive, DISCERN can be time-consuming and requires training for consistent use (49). In the context of video content, DISCERN has been adapted by some researchers to evaluate scripted narration or on-screen information, but its written-format origins may limit applicability to visual, interactive, or audiovisual cues that influence viewer perception and comprehension.

The JAMA Benchmark Criteria provide a more objective evaluation of online health information, assessing four elements: authorship, attribution, disclosure, and currency. While useful for gauging source credibility, the binary scoring system does not assess content accuracy, completeness, or audiovisual clarity—factors highly relevant in video-based media. As such, the JAMA criteria are often used as a supplemental tool rather than a standalone measure when evaluating videos (50).

The GQS criteria uses a 5-point Likert scale to assess the overall quality, flow, and usefulness of online content, especially videos, ranging from poor (1) to excellent (5). Though fast and intuitive, GQS is subjective and lacks detailed evaluative criteria, limiting its diagnostic utility (51). The HONcode certification, developed by the Health On the Net Foundation, was another credibility-focused tool that evaluated websites based on eight ethical principles, including authority, complementarity, privacy, attribution, justifiability, transparency, financial disclosure, and advertising policy. While HONcode was useful for identifying trustworthy health websites, it did not assess content depth, accuracy, or readability. As of December 15, 2022, the HONcode certification service has been discontinued, limiting its utility for future website evaluations (52, 53).

Eighty percent of articles rated the video educational quality as poor, suggesting a need to improve video quality. While these are established standardized tools, they are not designed to assess medical videos. These gaps suggest that the current tools are not entirely sufficient for ensuring high-quality educational content in dynamic online environments like YouTube (7). Therefore, a standardized tool should be created to assess video quality to ensure consistency across video evaluations.

In comparison to DISCERN, JAMA, and GQS, specialized frameworks such as the Instructional Videos in Otorhinolaryngology by YO-IFOS(IVORY) and LAParoscopic surgery Video Educational GuidelineS (LAP-VEGaS) guidelines have been developed to evaluate surgical videos intended for healthcare professional training (54, 55). These tools are more rigorous and procedure-specific in that they incorporate detailed criteria related to surgical technique, anatomical accuracy, intraoperative decision-making, step-by-step procedural clarity, video speed, camera angles, presentation clarity, and audio-visual delivery. Although these tools are designed for surgical training, they could be adapted to enhance the evaluation of surgical videos intended for patient education.

While the majority of the wide variety of assessment tools utilized by the articles indicated poor overall video quality, they also highlighted other problematic issues, including the omission of vital information, such as treatment alternatives, potential risks, low reliability scores, and increasing source bias. Several studies also pointed out technical issues, such as poor videography, suboptimal audio quality, and ineffective narration, which further detract from the educational value of the videos. Moreover, some studies identified misinformation and outdated content as critical problems, emphasizing the need for continuous updating and verification of online medical content. These deficiencies highlight another gap in the current use of YouTube as an educational tool for patients, and suggest that many videos fail to provide comprehensive information, which is essential for informed patient decision-making.

### Contrasting findings

Interestingly, three studies (8–10) rated the YouTube videos as adequate educational tools, citing high scores on quality assessment instruments, reputable sources, and clear, understandable content. This discrepancy indicates that while the general trend points toward inadequate educational quality, there are exceptions where videos meet high standards. These positive examples can serve as benchmarks for creating better educational content in the future.

## Limitations

This review is subject to several limitations. First, the included studies were assessed from the perspective of patients and healthcare consumers. While this approach is relevant to understanding public accessibility and perceived educational value, it may not fully reflect clinical accuracy or high educational quality. Additionally, it is important to acknowledge that YouTube functions primarily as an entertainment and social media platform rather than a formal educational resource. Consequently, many of the videos uploaded may not be intended for, or suitable as, educational content, limiting its suitability for patient and health consumer education. Another limitation is the exclusion of studies published in languages rather than English, which could introduce a potential selection bias, leading to incomplete or inaccurate conclusions, as studies published in other languages may contain crucial information that is not available in English-language sources.

### Future recommendations

This review highlights the need for improved standards in the creation of surgical educational videos on YouTube. A new standardized tool should be developed that incorporates the strengths of widely used current tools, while addressing the unique challenges of assessing medical videos. Key criteria should account for dynamic audiovisual elements (e.g., clarity of narration, visual accuracy of demonstrations, use of animations or overlays), content accuracy, source credibility, and viewer engagement strategies. It should also consider accessibility features such as closed captions, language simplicity, and cultural sensitivity.

Advancements in artificial intelligence (AI), particularly natural language processing and deep learning, present promising opportunities for moderating health-related video content. For example, real-time misinformation detection using machine learning has proven effective during the COVID-19 pandemic (56). To implement these innovations, we propose a multidisciplinary task force, composed of clinicians, AI researchers, digital media experts, public health officials, and patient advocates, to develop validated scoring systems and collaborate directly with platforms, such as YouTube. Integration strategies may include voluntary quality tagging by verified content creators, peer-review-based content badges, and platform-endorsed health information panels. These features may help elevate trustworthy content while guiding users toward evidence-based information in an increasingly decentralized and saturated media landscape.

By directing patients to high-quality educational resources, surgeons can significantly enhance patient understanding and preparedness for surgical procedures. Given the overwhelming amount of online medical information, surgeons must guide patients toward reputable websites, vetted educational videos, and institutionally approved resources. They should also be aware of the quality assessment tools available to evaluate the quality of video content, ensuring that the materials they endorse are of the highest standard.

## Conclusions

Though YouTube has indubitably transformed patient and health consumer education, the reliability and educational quality of its patient education videos remain a concern, particularly with surgical procedures. This systematic review finds that, despite their perceived credibility, quality assessment tools have determined that many videos from professional sources offer limited educational value. With improved patient education materials, the medical community can improve health consumer education, ultimately enhancing patient understanding, reducing anxiety, and potentially improving clinical outcomes in surgical settings.

## Data Availability

The original contributions presented in the study are included in the article/supplementary material, further inquiries can be directed to the corresponding author.
