# Supplementary material for: Tools/instruments for assessing YouTube videos on surgical procedures for patient/consumer health education: a systematic review
Source: Front Public Health. 2025 Jul 10;13:1575801. doi: 10.3389/fpubh.2025.1575801 (PMC12286933; doi:10.3389/fpubh.2025.1575801)
Supplement: Supplementary file 1 [file Data_Sheet_1.docx]

Appendix A:

A sample search strategy for PubMed:

"youtube"[Title/Abstract] AND (evaluat*[Title/Abstract] OR assess*[Title/Abstract] OR quality [tiab] OR scor*[tiab] OR tool* [tiab] OR measur*[tiab] OR instrument*[tiab] OR scale*[tiab] OR psychometrics OR reproducibility of Results [mh] OR reproducibility [tiab] OR reliability [tiab] OR validity[tiab]) AND ("Patient Education as Topic"[MeSH Terms] OR "information dissemination"[MeSH Terms] OR “health information” [tiab]  OR “health literacy” [tiab] OR “patient education” [tiab] OR “consumer health” [tiab]) AND (Surgical Procedures, Operative [mh] OR surgical procedure [tiab] OR surgical operation[tiab])
